# Supplementary material for: γδ T cell-mediated cytotoxicity against patient-derived healthy and cancer cervical organoids
Source: Front Immunol. 2023 Nov 27;14:1281646. doi: 10.3389/fimmu.2023.1281646 (PMC10711208; doi:10.3389/fimmu.2023.1281646)
Supplement: Supplementary file 1 [file DataSheet_1.docx]

Supplementary Material

γδ T cell-mediated cytotoxicity against patient-derived healthy and cancer cervical organoids

**Junxue Dong ^1,2^ *, David Holthaus ^1^ *, Christian Peters ^3^, Stefanie Koster ^2^, Marzieh Ehsani ^1^, Alvaro Quevedo-Olmos ^1^, Hilmar Berger ^1,2^, Michal Zarobkiewicz ^3,4^, Mandy Mangler ^5,6^, Rajendra Kumar Gurumurthy ^2^, Nina Hedemann ^7^, Cindrilla Chumduri ^2,8 #^, Dieter Kabelitz ^3 #^, Thomas F. Meyer ^1,2 #^**

**# Corresponding authors**

Prof. Dr. Thomas F. Meyer

Email: t.meyer@ikmb.uni-kiel.de

meyer@mpiib-berlin.mpg.de

Prof. Dr. Cindrilla Chumduri

Email: cindrilla.chumduri@bce.au.dk

Prof. Dr. Dieter Kabelitz

Email: Dietrich.Kabelitz@uksh.de

**Supplementary Figures**

**
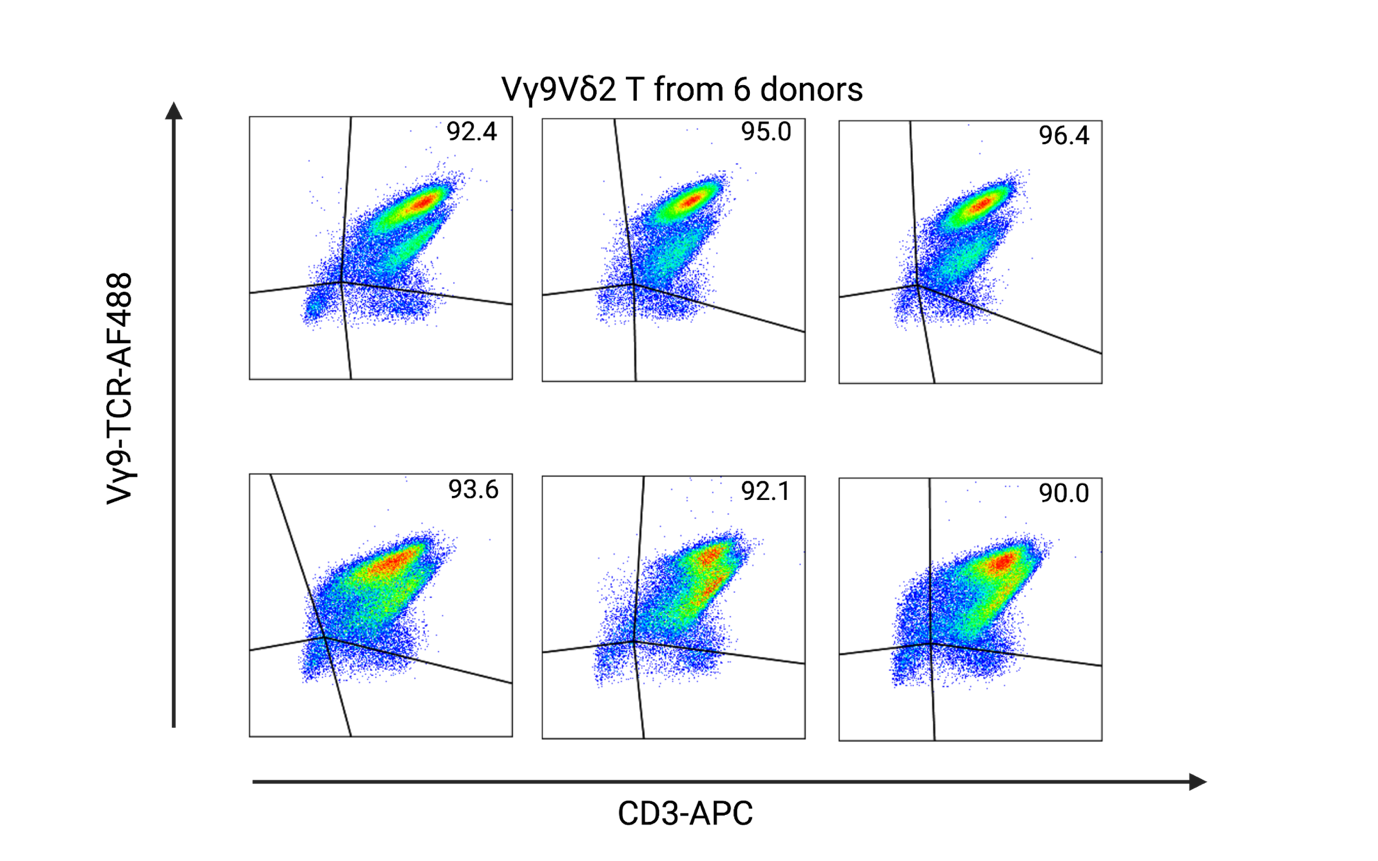
**

**Supplementary Figure 1: Exemplary purity measurements of γδ T cells by flow cytometry.** Only cultures with a purity >90% were included.


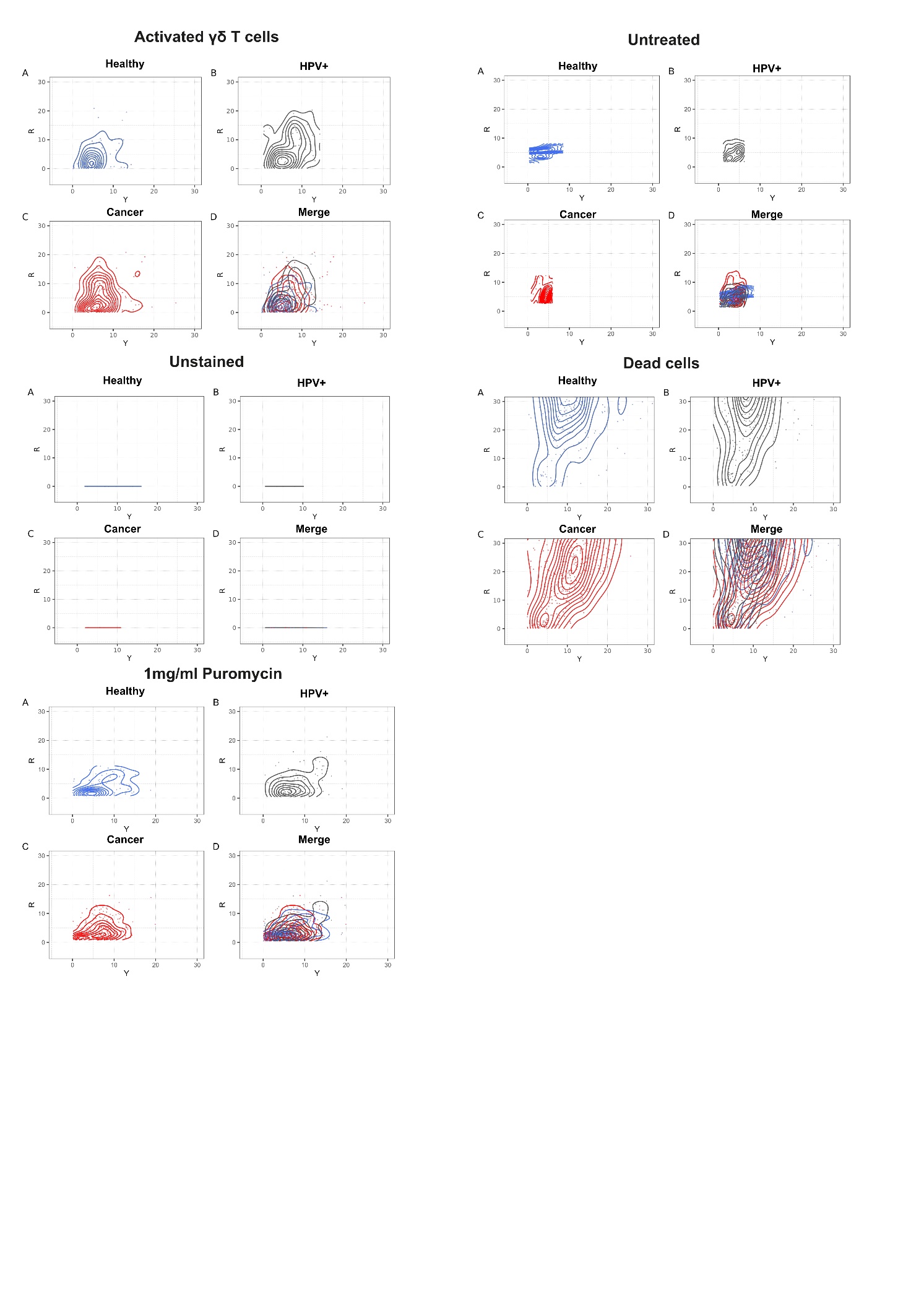


**Supplementary Figure 2: Scatter blots of live cell imaging depicting individual organoids after four hours in their respective conditions.** γδ T cells were activated with 300 nM BrHPP. Dead cells were boiled for 10 min at 95°C before start of the experiment, and 1 mg/ml Puromycin treated cells served as death control over time. Cells were stained with NucRed Live 647 Ready Probe Reagent. Yellow marks viable cells, red marks dead cells.


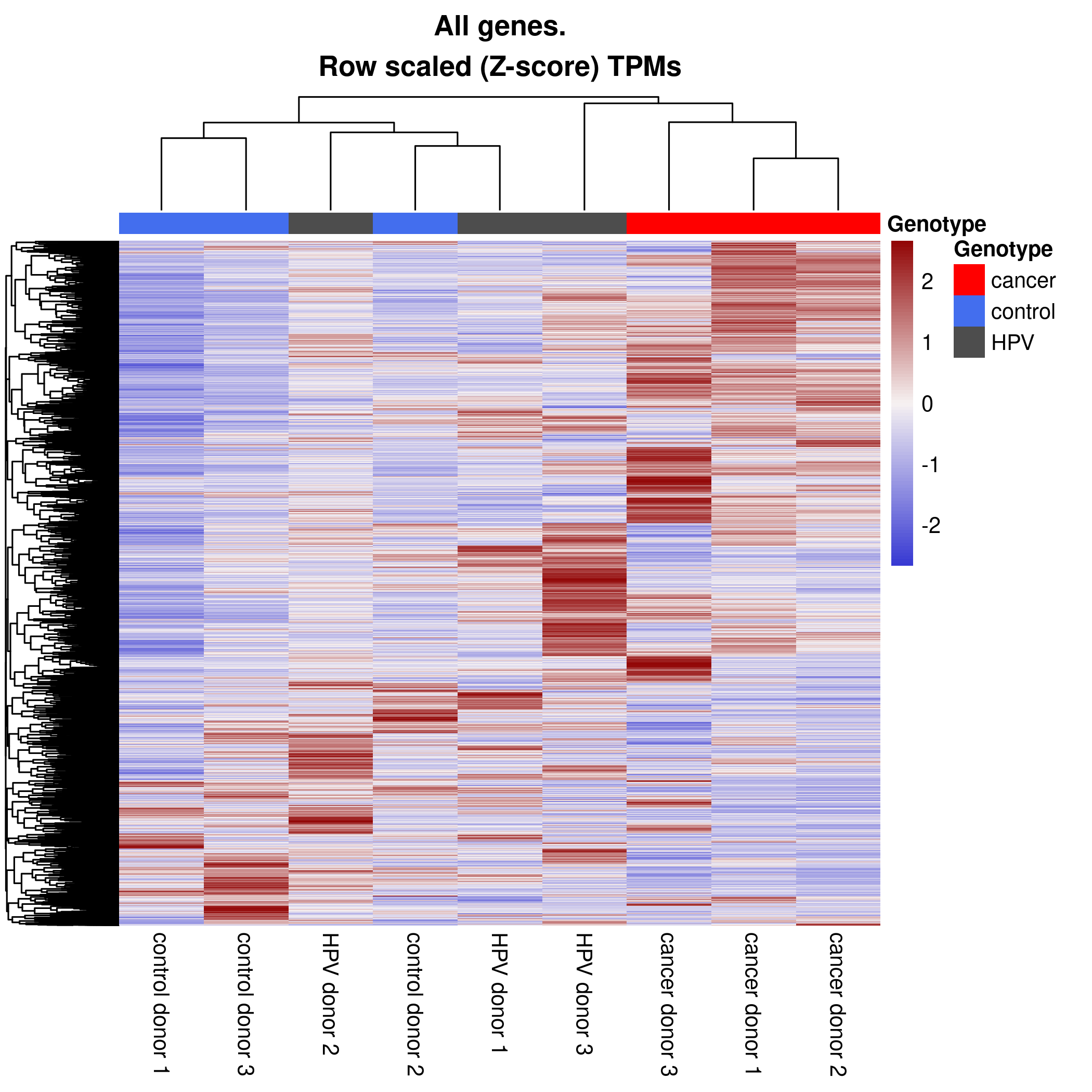


**Supplementary Figure 3:** **HPV16 integration alters organoid transcriptome***.* RNA sequencing was performed for 2 patient isolates per condition. Heatmaps of the z-score of normalized counts. Downregulated genes are marked in blue, and upregulated genes in red.


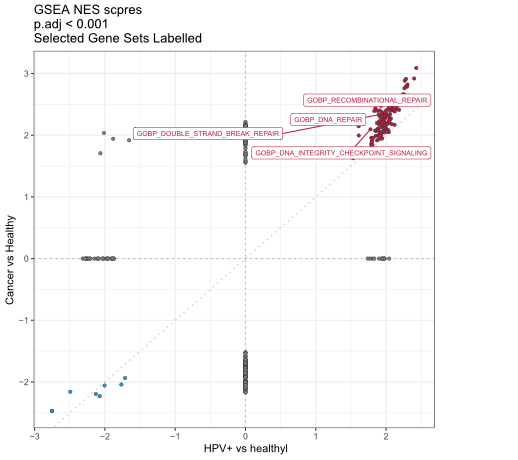


**Supplementary Figure 4: Significantly altered GOBP pathways as defined by GSEA analysis.** Exemplary DNA altering pathways are given. Only Pathways below p adj < 0.001 are shown.

**Supplementary Table 1: Antibodies used in the study.**

| **Target** | **Host** | **Vendor** | **Application** | **Dilution** |
| --- | --- | --- | --- | --- |
| **Primary antibodies** | | | | |
| KRT5-A488 | Rabbit | Abcam, ab193894, RRID:AB_2893023 | IF | 1:500 |
| KI67 | Rabbit | Abcam, ab16667, RRID:AB_302459 | IF  WB | 1:500  1:1,000 |
| BTN2A1 | Mouse | ImCheck Therapeutics, mAb 7.48, | Blocking | 5 µg/ml |
| BTN3A1/A2/A3 | Mouse | ImCheck Therapeutics, mAb 103.2 | Blocking | 5 µg/ml |
| MSH2 | Rabbit | Cell Signaling, 2017S, RRID:AB_2235387 | WB  Blocking | 1:1,000  3 µg/ml |
| CD107a-PE | Mouse | BD Biosciences, Clone H4A3, 560948, RRID:AB_10565964 | FC | 2.5 µg/ml |
| TCR Vδ2-FITC | Mouse | Beckman Coulter, Clone B6, 555738, RRID:AB_396081 | FC | 2 µg/ml |
| TCR Vγ9-FITC | Mouse | Conjugated in house, Janssen et al. (1991) | FC | 1:20 |
| CD3-APC | Mouse | BD Biosystems, Clone UCHT1, 561810, RRID:AB_10893350 | FC | 2 µg/ml |
| HLA-ABC-PE | Mouse | BD Bioscience, 555553, RRID:AB_395936 | FC | 1:20 |
| PD-L1/CD274  PD-L1/CD274-APC | Rabbit  Mouse | Cell Signaling, 13684S, RRID:AB_2687655  Invitrogen, 17-5983-42, RRID:AB_10597586 | WB  FC | 1:1,000  1:20 |
| ACTB | Mouse | Sigma, A5441, RRID:AB_476744 | WB | 1:10,000 |
| **Secondary antibodies** | | | | |
| Anti-Rabbit A647 | Donkey | Jackson ImmunoResearch, 711-605-152, RRID:AB_2492288 | IF | 1:100 |
| Anti-Rabbit HRP | Goat | Cell Signaling, 7074S, RRID:AB_2099233 | WB | 1:3,000 |
| Anti-Mouse HRP | Horse | Cell Signaling, 7076S, RRID:AB_330924 | WB | 1:3,000 |

**References:**

Janssen, O., Wesselborg, S., Heckl-Ostreicher, B., Pechhold, K., Bender, A., Schondelmaier, S., Moldenhauer, G., and Kabelitz, D. (1991). T cell receptor/CD3-signaling induces death by apoptosis in human T cell receptor gamma delta + T cells. *J Immunol* 146**,** 35-39.
